# Energetics of the protein-DNA-water interaction

**DOI:** 10.1186/1472-6807-7-4

**Published:** 2007-01-10

**Authors:** Francesca Spyrakis, Pietro Cozzini, Chiara Bertoli, Anna Marabotti, Glen E Kellogg, Andrea Mozzarelli

**Affiliations:** 1 Department of Biochemistry and Molecular Biology, University of Parma, viale Usberti 23/A, 43100 Parma, Italy; 2 Laboratory of Molecular Modeling, Department of General and Inorganic Chemistry, University of Parma, Viale Usberti 17/A, 43100 Parma, Italy; 3 Laboratory for Bioinformatics and Computational Biology, Institute of Food Science, National Research Council, Via Roma 52 A/C, 83100 Avellino, Italy; 4 Interdepartmental Research Center for Computational and Biotechnological Sciences (CRISCEB), Second University of Naples, Via S. Maria di Costantinopoli 16, 80138 Naples, Italy; 5 Department of Medicinal Chemistry & Institute for Structural Biology and Drug Discovery, Virginia Commonwealth University, Richmond, Virginia, 23298-0540, USA

## Abstract

**Background:**

To understand the energetics of the interaction between protein and DNA we analyzed 39 crystallographically characterized complexes with the HINT (Hydropathic INTeractions) computational model. HINT is an empirical free energy force field based on solvent partitioning of small molecules between water and 1-octanol. Our previous studies on protein-ligand complexes demonstrated that free energy predictions were significantly improved by taking into account the energetic contribution of water molecules that form at least one hydrogen bond with each interacting species.

**Results:**

An initial correlation between the calculated HINT scores and the experimentally determined binding free energies in the protein-DNA system exhibited a relatively poor r^2 ^of 0.21 and standard error of ± 1.71 kcal mol^-1^. However, the inclusion of 261 waters that bridge protein and DNA improved the HINT score-free energy correlation to an r^2 ^of 0.56 and standard error of ± 1.28 kcal mol^-1^. Analysis of the water role and energy contributions indicate that 46% of the bridging waters act as linkers between amino acids and nucleotide bases at the protein-DNA interface, while the remaining 54% are largely involved in screening unfavorable electrostatic contacts.

**Conclusion:**

This study quantifies the key energetic role of bridging waters in protein-DNA associations. In addition, the relevant role of hydrophobic interactions and entropy in driving protein-DNA association is indicated by analyses of interaction character showing that, together, the favorable polar and unfavorable polar/hydrophobic-polar interactions (i.e., desolvation) mostly cancel.

## Background

Macromolecular recognition is based on the requirement of dual geometric and chemical complementarity, eventually leading to the formation of a thermodynamically stable and specific complex between interacting molecules. These aspects are key elements for understanding the function of biological systems: enzymes that bind substrates and effectors, proteins that mediate signal transduction via networks of alternative or specific protein-protein pair, and nucleic acids that, via the binding of transcription factors, repressors, co-activators, regulate protein expression. In particular, the site-specific associations between DNA and proteins regulate most biological events [1], with key involvement in transcription, replication and recombination. Matthews [2] stated "the full appreciation of the complexity and individuality of each complex will be discouraging to anyone hoping to find simple answers to the recognition problem". A few years later, Draper [3] was still asking "...how does a protein select a specific DNA site out of the many available, when all potential binding sites share such a high degree of structural similarity? Thermodynamic, as well as structural, approaches must be used to answer this question... ". Now, more than a decade later, no simple model for recognition between amino acids and nucleotides has been found [2,4-7]. From the analysis of the first protein-DNA crystal structure it was evident that several distinct contributions lead to formation of the complex [8-10], i.e., hydrogen bonds, electrostatic interactions, direct and indirect contacts between amino acids and phosphate, sugars and bases, water-mediated contacts, hydrophobic effects, ion release, mutual conformation rearrangement, bending and distortion. Amongst the enthalpic contributions, hydrogen bonds are the most easily recognized, and energetically may represent the bulk of interactions between nucleic acids and proteins, comprising protein backbone and side-chains contacting bases at their edges and the polynucleotide backbone [11]. About half of the hydrogen bonds found in known protein-DNA complexes involve phosphodiester oxygens [12], initially mediating indirect recognition between DNA and protein, and favoring a subsequent localization of the protein in a specific site [13]. In direct recognition, representing the foundation of sequence specificity, hydrogen bonds are formed between amino acid side-chains and DNA bases. Even earlier in the binding process, entropy plays a significant role in recognition as non-specific (low affinity) interactions, driven by long-range electrostatic forces, bring the DNA and protein into proximity and cause the release of counter-ions from the free DNA [14]. Thusly, water molecules in free and protein-bound DNA complexes have been thoroughly investigated both experimentally and theoretically, and different roles have been proposed for interaction and recognition (see [14-16] and references therein).

While enthalpy is associated with molecular interactions resulting from complex formation, entropy is associated with multiple protein and DNA conformations, variations in the structure of water molecules and counterions, and other factors. This complexity and the interplay between multiple chemical and physical mechanisms necessary to achieve the required level of specificity are extremely difficult to describe quantitatively [17,18]. Recent investigations using osmotic pressure [19] has led to a determination of the differential role and number of water molecules released in specific and nonspecific binding of protein to DNA sequences [20,21]. Some results from these studies do not appear to be supported by x-ray crystallographic data of specific and nonspecific protein-bound DNA complexes [15]. Interestingly, osmotic pressure experiments suggest that *in vitro *studies in dilute solutions are likely to be less informative on *in vivo *processes than expected, due to the presence of crowding and confinements effects [22,23]. This, in turn, implies that the biological environment is relatively similar to that experienced by macromolecules within a crystal lattice [15,24,25]. Computational methods, which are heavily dependent on x-ray crystallographic data and are widely and successfully used in the evaluation of the energetics of ligand-protein interactions [26-29], should also be applicable to understanding protein-DNA complex formation. The earliest attempts [3,30] tried to estimate the contribution of each pair of amino acid residues/nucleotide bases with respect to the total protein to DNA binding affinity. A different approach, proposed by Mandel-Gutfreund and Margalit [5], assumed that a global score reflecting the complementarity between a protein and its DNA target can be calculated by statistical analyses of the frequency of interactions for specific amino acid residue-nucleotide base types, thus implying additivity in binding energetics. Other attempts to qualitatively, semi-quantitatively and/or quantitatively describe the interaction between protein and DNA [6,7,11,14,31-37], have taken advantage of the available three-dimensional crystallographic structures of proteins that bind to DNA, a field pioneered by Matthews [38].

A wealth of information on the rules that govern biomolecular recognition is derived from structural data, predominantly x-ray crystallography and nuclear magnetic resonance. However, the analysis of the three-dimensional structure of a complex can only provide a geometric framework that ultimately needs quantitative evaluation of the binding energetics to enable assessment of codes, rules, and/or mechanisms. To date, pursuit of this goal has primarily focused on ligand-protein interactions due to the intense interest in designing compounds that bind selectively and with high affinity to therapeutically relevant enzyme or protein targets. Consequently, a variety of computational modeling approaches have been developed to obtain quantitative descriptions of ligand-protein encounters. Usually, the process is simplified by: i) considering only the volume specified by the active site; ii) assuming no or reduced conformational flexibility; and iii) neglecting the energetic contributions of water molecules (both with respect to their contribution to enthalpy and entropy).

To overcome some of these limitations, the HINT (**H**ydrophatic **INT**eractions) force field was developed. HINT is based on LogP_o/w_, the solvent partition coefficient of a species between 1-octanol and water, these solvents being models for the internal apolar and polar protein milieus, respectively [39]. Because LogP_o/w _is a free energy parameter, its measurement takes into account both enthalpic and entropic contributions originating from all molecules, including water, that participate in a biomolecular association, and solvent partitioning data are unique experimental measurements of intermolecular and interatomic interactions. The total interaction score (**B**) for a complex is calculated with the following equation:

**H_TOTAL _**= **Σ_i_Σ_j_b_ij _**= **Σ_i_Σ_j_(a_i _S_i _a_j _S_j _T_ij _R_ij _+ r_ij_)**,     (1)

where **b_ij _**represents the interaction score between atoms **i **and **j**, **a **the atomic hydrophobic atom constant, **S **the atomic solvent accessible surface area, **T_ij _**a logic function assuming +1 or -1 values, depending on the polar nature of interacting atoms, and **R_ij _**and **r_ij _**are, respectively, functions of the distance between the atoms i and j. **R_ij _**is usually a simple exponential function, while **r_ij _**is an adaptation of the Lennard-Jones function. The key parameters **a **are calculated by a procedure adapted from the CLOG-P method [40]. Because the sum of all **a_i_**s is the LogP_o/w _for a molecule, each **a_i _**is a partial logP that can be considered a δg for solute transfer. If the "receptor" is changed from the 1-octanol/water solvent pair to a biomacromolecule with hydrophobic and polar regions, then, in a sense, the **a_i_**s represent atomic free energies of association. Each **a_i _**thus encodes all aspects of free energy, both enthalpic and entropic. In HINT, favorable **b_ij _**interactions (hydrogen bonds, acid-base, hydrophobic-hydrophobic) are positively scored, while unfavorable contacts (acid-acid, base-base, hydrophobic-polar) are negatively scored in the HINT paradigm. **H_TOTAL_**, the sum of all **b_ij _**terms describes the total interaction between the two species. In this way, the ligand-protein interaction is not separated in multiple factors by interaction type (e.g., hydrogen bond, hydrophobic, etc.), but is considered a concerted event, as it occurs in nature [41]. Because the HINT analysis is carried out on biomolecular systems with three-dimensional structure, geometric information is embedded in the procedure. We have applied this approach to the energetics of protein-ligand complexes both in the absence and presence of water molecules that bridge protein and ligand, at constant pH and as a function of the ionization state of interacting groups [29,42-44], in protein-protein interfaces [45,46], and in ligand-DNA recognition [47-49]. Results from these diverse studies indicate that HINT is a powerful tool to quantitatively investigate and describe the energetics and specificity of biomolecular processes. It must be noted that HINT analysis evaluates the interactions between pre-formed molecules, and does not include terms for evaluating the internal energies of these molecules. These internal energies are certainly important components of overall binding free energy, but may be relatively invariant within a particular data set as we have reported [29,42-49].

In the present work HINT analysis was used to evaluate the strength of interaction in protein-DNA complexes, explicitly taking into account the energetic contribution generated by water molecules found at the interface between protein and DNA. This analysis was performed on 39 DNA-protein complexes, determined at resolution better or equal to 2.8 Å and for which experimental equilibrium constants are available. Correlation of HINT score with experimental free energy indicates predictive models with a standard error of ± 1.28 kcal mol^-1^. These results represent a quantitative basis for ultimately dissecting the amino acid residue-nucleotide base interaction to understand the amino acid-base "recognition code", a topic we are currently investigating.

## Results and discussion

Being able to accurately model the energetics of protein-DNA association will help us to more completely understand the machinery of life itself and to uncover a wealth of new opportunities for the therapeutic treatment of many diseases. While direct interactions, i.e., recognition, between the two macromolecules are important for specificity, the water molecules at protein-DNA interface also contribute to the complex formation and potentially play a role in mediating specific interactions (see [14-16] and references therein). In fact, Janin reports that protein-protein and protein-DNA interfaces contain at least as many water-mediated interactions as direct hydrogen bonds or salt bridges [50]. Water molecules mediating biological interactions have been the subject of intense recent study [16,43,44,51-53]. The importance of water in regulating recognition, complex formation and, generally, interactions among biomolecules is widely accepted, but experimental and computational tools for quantifying these effects are still somewhat lacking [54]. Even x-ray crystallography at high resolution likely underestimates the number of solvent molecules, and can misrepresent other ions, precipitant molecules or artifacts as water. One approach we have previously used to validate crystallographic water sets is application of the GRID program [55], which evaluates empty regions of space in terms of water (or another probe) being favorably bound. We found that crystallographic water molecules with high specificity virtually always exhibit favorable GRID energies [43], and thus should be considered well-placed.

Both protein and DNA molecules in solution, when uncomplexed, are surrounded by a variable number of water molecules interacting through hydrogen bonds with exposed polar groups. While the protein solvation pattern is extremely variable, a consequence of the protein's nature and folding [18,56], DNA presents largely the same (conserved) hydration pattern, with minor sequence-dependent local variation. An ordered spine of hydration occupies the minor groove, whereas the major groove is too wide to retain the same water network and is filled with ordered water molecules interacting singly or in pairs with the nucleotide bases [15]. In addition, the phosphate groups are usually surrounded by six hydration sites, with positions differing with the conformation and nucleotide types [57]. Overall, these conserved water patterns contribute to stabilization of the DNA conformation [16].

**Figure 1:**
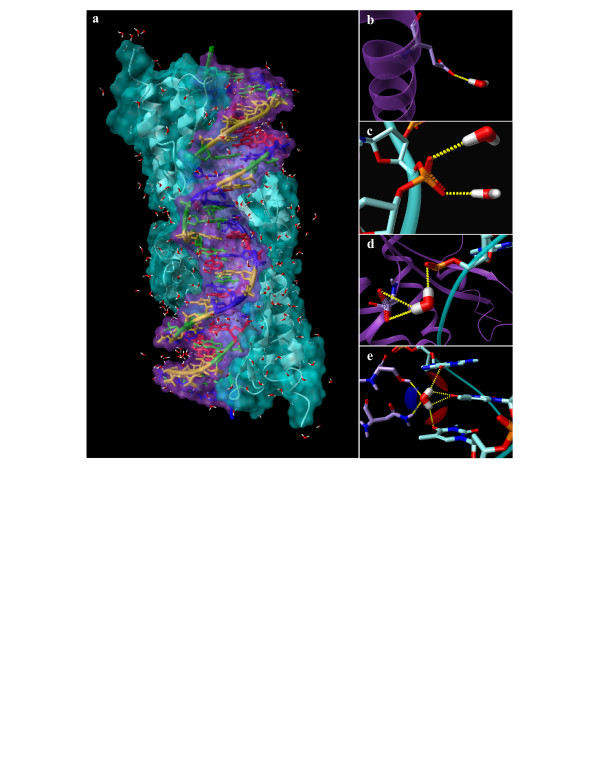
Three-dimensional structure representation of Homing endonuclease I-crei complex (1g9y), using display features of the Lithium software package [75]. **a) **Overall view of the complex where the protein is displayed in ribbon/tube style and the DNA is represented in color-coded ribbons: red for adenine (A), blue for cytosine (C), green for guanine (G), and yellow for thymine (T). **b) **Water molecule hydrating a negatively-charged amino acid side-chain. **c) **Water molecule hydrating a DNA phosphate group. **d) **Water molecule screening the repulsive interaction between an Asp side-chain and a DNA phosphate. **e) **Water molecule located at the complex interface mediating specific amino acid-base interactions.

The process of protein-DNA association is certainly very complex, with substantial conformational changes of the interacting macromolecules and a concomitant significant rearrangement of the bound solvent water molecules and counterions. Matthews, in his pioneering work on protein-DNA interaction, recognized the fundamental role played by water molecules in mediating the formation of the Trp repressor-DNA complex, stating that "the explicit need to consider bound water on the surfaces of both proteins and DNA adds another level of complexity to the recognition problem" [2]. Figure 1a illustrates the three-dimensional structure of the homing endonuclease I-Crei complex (PDB code 1g9y), one of the 39 complexes studied in this work, including display of the bound water molecules. Water molecules hydrating exposed polar groups on the protein and DNA respectively, are highlighted in Figure 1b and 1c. The goal of this paper is to unravel the energetics of association as they relate to recognition between protein and DNA. We will put particular emphasis on understanding the contribution and role of water in protein-DNA associations.

### Protein-DNA interaction energetics

**Figure 2:**
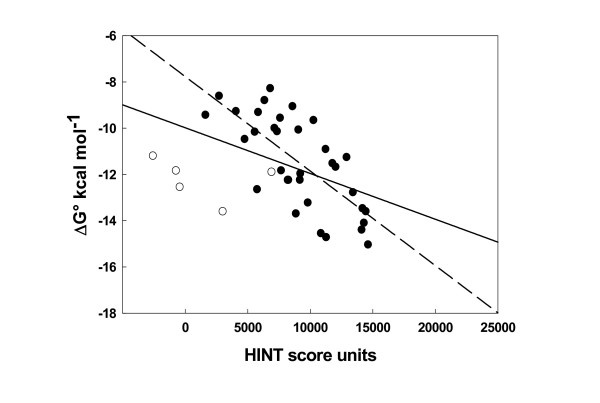
Correlation between the experimental binding free energies and the calculated HINT score values for the 39 analyzed protein-DNA complexes. Solid line is correlation for all 39 complexes; dashed line is correlation after five endonuclease I-CreI-DNA native and mutant complexes (1g9z, 1g9y, 1t9i, 1t9j, 1u0c; open symbols) are removed from dataset.

The structures of 39 proteins bound to their target DNA sequences were retrieved from the Protein Data Bank [58] and from the Nucleic Acid Database [59] (see Table 1). In order to obtain reliable calculations and predictions, only structures characterized by resolution better than 2.90 Å were considered: the average resolution is 2.18 Å. Other selection criteria are described in the Methods. The data set is composed of DNA binding proteins with different functions, i.e., 6 transcription factors, 19 transcription regulators, 12 enzymes and 2 DNA binding proteins. The binding affinities for the complexes vary over about four orders of magnitude. The interaction of each protein with its corresponding DNA sequence was evaluated with the HINT force field [39] (Table 2). Correlation (Figure 2, solid line) of the calculated HINT scores for each protein-DNA association with the experimentally determined free energies of binding for that complex (all symbols) leads to:

ΔG° = -0.000198 H_TOTAL _-9.98,     (2)

with a relatively poor r^2 ^= 0.21 and a standard error of ± 1.71 kcal mol^-1^. However, several outliers (open symbols) are evident, negatively affecting the correlation. All outlier complexes contain the same protein: homing endonuclease I-CreI, complexed in the native form with either the DNA product (1g9z) or the DNA substrate (1g9y), and enzyme mutants (1t9j and 1u0c). While the data point for the endonuclease I-CreI substrate complex 1t9i is well placed in this correlation, it is considered an outlier in this discussion (vide infra). The exclusion of these five outliers produces a significantly improved correlation (Figure 2, dashed line):

ΔG° = -0.000409 H_TOTAL _-7.77,     (3)

with an improved r^2 ^of 0.51 and a decreased standard error of ± 1.41 kcal mol^-1^.

The count of solvent molecules is extremely variable in the analyzed structures (Table 2), ranging from 2 in 1jkr to 857 in 1g9z, with a mean value of 200. We have shown previously that water molecules, in particular those that bridge between interacting species, play a significant energetic role in biomolecular associations [43]. Significantly, the average number of crystallographically detected waters in the endonuclease I-CreI-DNA complexes (1g9z, 1g9y, 1t9i, 1t9j, 1u0c) is much higher, 454. Complexes with an overall high number of crystallographic waters would also be expected to have a concomitantly high number of potentially bridging and energetically relevant waters at the protein-DNA interface. Since a high water count in crystallographic models is usually due to higher accuracy in the x-ray structure as a larger fraction of bound waters are revealed, the crystallographic resolution of the five endonuclease I-CreI-DNA complexes (varying between 1.6 to 2.5 Å with an average of 1.99 Å), is only a partial cause of this difference. It is important to note, however, that water molecules may be introduced during crystallographic refinement only to account for electron density with unknown origin, which improves the apparent data analysis statistics.

### Water role in protein-DNA interaction energetics

**Figure 3:**
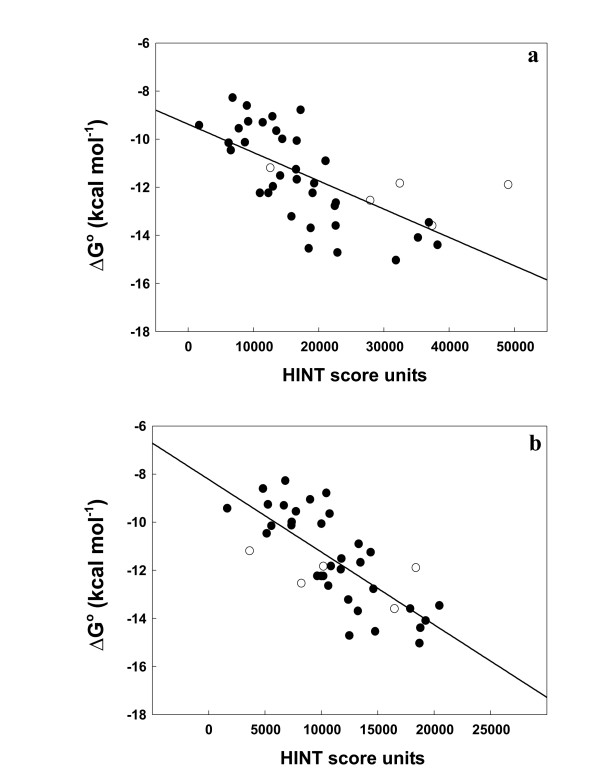
Correlation between the experimental binding free energies and the calculated HINT score values taking into account the score contributions of water molecules. **a) **Including all water molecules within in a 4 Å range of both atoms of the protein and atoms of the DNA. **b) **Including waters in a 4 Å range with total Rank ≥ 4, and non-zero partial Ranks with both protein and DNA.

Water can play several fundamental roles at the interface of protein-DNA systems [16]. Water molecules can: i) fill destabilizing holes in the complex; ii) facilitate binding by screening unfavorable electrostatic contacts (Figure 1d); and iii) act as linkers or "bridging waters" at the protein-DNA interface by providing side chain "extensions" that facilitate indirect hydrogen bonding (Figure 1e). We evaluated the contribution of water molecules placed at the protein-DNA interface by first identifying all waters (oxygen atoms) that are ≤ 4 Å from both protein and DNA. This set contains an overall total of 1244 water molecules (Table 3). When, as described in Material and Methods, the contribution of these interfacial waters was added to the protein-DNA HINT score, the correlation of H_TOTAL _with the experimental free energy of association (Figure 3a), is described by the following equation:

ΔG° = -0.000118 H_TOTAL _-9.38,     (4)

with r^2 ^of 0.43 and standard error of ± 1.45 kcal mol^-1^. Complexes previously identified as outliers (Figure 2) are now coherent with the correlation, supporting the fundamental contribution played by water molecules to the free energy of binding between protein and DNA. Only 1t9i, the endonuclease I-CreI-DNA complex that was not an obvious outlier in Figure 2 (but nevertheless removed), is an obvious outlier in Figure 3a.

Previous analyses of protein-ligand systems indicated that only "bridging" water molecules are relevant for complex formation [43], and these highly constrained waters should be located in crystallographic experiments of even moderate resolution. We used the Rank algorithm [60], which has been validated with a wide set of protein and protein-ligand structures [44], to identify bridging waters and predict the weighted number of hydrogen bonds potentially formed by each with both the protein and the DNA. Using the filter that only waters characterized by Rank greater than 0 with both macromolecules (i.e., forming at least one hydrogen bond with each) are included in H_TOTAL_, the number of significant waters placed at the protein-DNA interface is reduced from 1244 to 996 (Table 3) for all complexes. Correlating this H_TOTAL _with free energy (not shown) yielded a model with r^2 ^of 0.47 and standard error of ± 1.41 kcal mol^-1^. Visual inspection suggested, however, that some of the members of this water set are not *truly *bridging, possibly because the Rank algorithm does not distinguish between distance and angular contributions to Rank. The implication is that Rank only modestly greater than zero may correspond to unstable and very weak contacts.

Our previous studies of water molecules in proteins and protein-ligand complexes [44] demonstrated that water molecules with total Rank of at least 4 and non-zero partial Ranks had more impact on the formation of protein-ligand complexes. Waters with Rank ≥ 4 should form at least two hydrogen bonds and have very favorable geometry and thus be more locked and stable at the protein-DNA interface and, thus, more detectable by X-ray diffraction analysis. The number of waters that satisfy these criteria is 261. The more "fixed" position of these waters is confirmed by a relatively lower mean B factor (32.7 Å^2^) than the mean value calculated for all the 7394 crystallographic water molecules (42.8 Å^2^). Correlation of H_TOTAL _calculated with this set of waters with free energy (Table 2 and Figure 3b) yields:

ΔG° = -0.000302 H_TOTAL _-8.22,     (5)

with r^2 ^= 0.56 and standard error ± 1.28 kcal mol^-1^. The improvement in the correlation is clearly due to the contributions of a smaller, but more significant, set of water molecules. The 261 waters, in fact, correspond to just 3.5% of the whole set of crystallographically detected water molecules in the 39 complexes. This value is close to the 5.5% identified by Reddy and co-workers as the percentage of waters contacting simultaneously both the protein and the DNA and thus mediating recognition directly, on a set of 17,963 analyzed crystallographic water molecules [51]. This model has no outliers: the reduction in the count of potential bridging waters from 98 to 31 in the 1t9i complex led to a considerable decrease in H_TOTAL _from 43,140 to 18,371, positioning this point within about 1.5 kcal mol^-1 ^of the correlation line. The water contribution to the total interaction energy for all complexes is 28%. But, removing the endonuclease I-CreI-DNA native and mutant complexes from the data set, where the solvent contribution to the overall binding process is seemingly anomalously high, reduces the water contribution to just 16% of H_TOTAL _for the remaining 34 complexes. This value is similar to the fraction of water-mediated bonds (14.9%) estimated by Luscombe and Thornton [61] after a geometry-based analysis of all protein-DNA interactions.

It is important to emphasize that the results presented here explain only part of the protein-DNA-water interaction and the tools we have used only illuminate the process through examination of the bound endpoint. For example, the energetic contribution of the internal conformations, i.e., conformational entropy, of the interacting biopolymers is not treated explicitly, and is only one of several components of the additive constant portion of our correlations (eqs. 2–5). However, the low standard errors in our models indicate that these contributions are more or less constant across the data series. The magnitude of the additive constant can be rationalized by the fact that these complexes *do *have many structural and chemical similarities – the most important of which is that they all form crystals analyzable by x-ray diffraction. Note (eqs. 2, 4 and 5) that as we incrementally improved the models by explicitly including more appropriate sets of water molecules, the additive constant decreased in magnitude as the standard error improved, indicating that this particular contribution to ΔG° is now being treated more explicitly.

### Energy contributions of the DNA base, phosphate and ribose to complex formation

The association of a protein-DNA complex usually involves a two-step process: an initial binding via non-specific interactions and a subsequent translocation of the protein to the specific binding site [62,63]. The first step is regulated by electrostatic contacts between the protein side-chains and the DNA backbone phosphates, while binding specificity is achieved by interactions with the nucleotide bases themselves. However, the DNA backbone (ribose and phosphates) may play a less dramatic but fundamental role in specificity by holding the protein in a defined orientation, thus decreasing the energetic cost of the complex formation, or because the phosphate orientations are somewhat determined by the base sequence [12]. From a geometric-based analysis, which evaluated two atoms to be in contact if their centers were 1–5 Å apart, Lejeune and co-workers [64] reported that an average of 47% protein-DNA interactions involve the phosphate group, while 24% can be attributed to the base.

**Figure 4:**
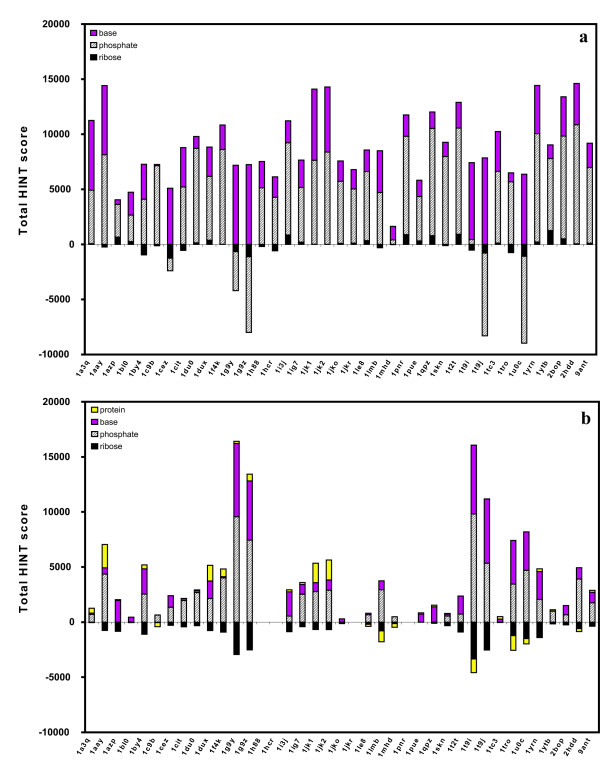
**a) **Contributions of various structural components of the protein-DNA association. Shown are protein-DNA_phosphate_, protein-DNA_ribose _and protein-DNA_base-edge _contributions, colored as indicated in the legend. **b) **HINT score contributions calculated for the interaction between water molecules (having total Rank ≥ 4 and non-zero Rank with both macromolecules) and the protein-DNA interface. Shown are the water-DNA_phosphate_, water-DNA_ribose_, water-DNA_base _and water-protein contributions, colored as indicated in the legend. Bars corresponding to 1h88, 1hcr, 1jkr and 1pnr complexes are missing because no significant crystallographic water molecules were found in the crystal structures.

The total HINT score for each analyzed complex was deconvolved into partial contributions from protein-DNA_phosphate_, protein-DNA_ribose _and protein-DNA_base _interactions (Figure 4a). Clearly, both the non-specific (DNA backbone) and the specific contacts (base) play a fundamental role in driving the association event and in stabilizing the complex. The protein-DNA_phosphate _HINT score values are variable, ranging from -7900 to 10800 HINT score units (Table 2), but the most negative contributions are found in the endonuclease I-CreI-DNA complexes 1g9y, 1g9z, 1t9j and 1u0c, noted for their non-conforming behavior earlier. These high negative-valued protein-DNA_phosphate _contacts clearly explain the low overall protein-DNA HINT scores calculated for complexes 1g9z, 1t9j and 1u0c (Table 2), whose association seems unfavorable without a compensating water term. In these cases, water molecules do not only mediate the recognition between amino acids and bases, but may also significantly screen the unfavorable electrostatic interactions between phosphate groups and negatively charged amino acid side-chains. Thus, the high number of water molecules is needed to stabilize the DNA/I-CreI endonuclease complexes. The ribose groups do not significantly participate in the binding energetics (Figure 4a) and, in fact, the protein-ribose interactions are equally likely to be favorable or unfavorable with respect to HINT score. In contrast, the DNA bases contact the protein with ubiquitously favorable interactions (mean value of 3300 HINT score units). It is important to note that recognition is a complex process; for example, there is an almost complete absence of protein-DNA_base _interactions in the specific complex between TFIIBc and its target DNA sequence (1c9b), so this recognition must be mediated by protein-DNA_phosphate _or protein-DNA_ribose _interactions [12].

Both direct, including hydrophobic interactions, and indirect (water-mediated), interactions between the protein and DNA are relevant [12,64]. Figure 4b illustrates the contribution of the 261 interfacial (bridging) water molecules (both water-protein and water-DNA partial Ranks > 0, total Rank ≥ 4, as in eq. 5) to the protein-DNA interaction, where the water-DNA_phosphate_, water-DNA_ribose_, water-DNA_base _and water-protein terms are individually shown. The favorable DNA-water interaction is generally attributable to both water-DNA_phosphate _and water-DNA_base _contacts, reinforcing the notion that in most complexes water facilitates binding by screening unfavorable electrostatic contacts and acts as a linker at the protein-DNA interface. The water-DNA_phosphate _HINT score ranges from near zero to 9800 with a mean value of 2400, while the water-DNA_base _contribution ranges from near zero to 6700 HINT score units, with a mean value of 1600. Only in a few cases is a positive DNA-water HINT score completely attributable to the water-DNA_base _interaction; e.g., in 1azp, 1bl0, 1pue and 1qpz complexes water mediation is necessary to achieve specific recognition between the two macromolecules. The water-DNA_ribose _interaction always negatively affects the global HINT score because of unfavorable hydrophobic-polar contacts made between water and the hydrophobic moieties of ribose. Finally, the score contributions from protein-water contacts range from -1340 to 2120, with an average of only 140 units. The discrepancies between protein-water and DNA-water HINT scores will be discussed later, but are generally attributable to the different chemical natures of the interacting groups. It is evident in comparing Figure 4a and 4b that in the cases where the overall protein-DNA score is negative (i.e., the DNA/I-CreI endonuclease complexes), the water terms are able to compensate.

### Water molecules in protein-DNA interaction specificity identified by role

Coordinating water molecules are found in high numbers around protein-DNA complexes, and they play a variety of roles in stabilizing these complexes. To ascertain these roles, and to determine if conclusions regarding role could be determined by their Rank, we visually analyzed the larger set of waters; i.e., the 996 water molecules having non-zero Rank with respect to both macromolecules. Of these, somewhat more than half (547) interact with phosphate and ribose groups of the DNA in the ways described above. The main role of these waters is to screen unfavorable electrostatic forces arising between phosphate groups and charged amino acids side-chains. These waters are tightly bound to the DNA, with an average partial HINT score (H_DNA(backbone)-water_) of 426 (Table 4), but a weakly negative H_protein-water _of -36. On the other hand, the corresponding Rank has the opposite trend, the partial Rank for protein (1.9) is larger than that for the DNA (1.3). Together these data suggest that these waters are predominantly locked by a *single *very strong interaction with the DNA, and that favorable interactions between the protein and the DNA backbone are not actually mediated by water to any large extent other than by shielding the highly negative phosphate charge and generally making the environment around the phosphates more conducive to protein binding.

The remaining 461 waters of the set interact only with the bases of the polynucleotide. Each was then categorized (Table 4) as either non-bridging (when they are positioned such that cannot link protein and DNA base or when they unnecessarily mediate already favorable interactions between protein and DNA bases) or bridging (mediating specific protein-DNA recognition and association). This analysis identified 212 waters as non-bridging with an average Rank of 2.8. In fact, only 23 of these non-bridging waters (10%) have Rank ≥ 4. Among the 249 nucleotide base-to-protein bridging waters, 218 are found between bases and amino acid side-chains, 20 between bases and protein backbone, and 11 connect the bases to both the side-chain and backbone of the protein. The average Rank of these bridging waters is 3.7, with those linking to both the protein side-chain and backbone having an unsurprisingly larger average Rank of 4.6. One-third (82) of the bridging waters have Rank ≥ 4. HINT scores and Rank statistics for the set of waters interacting with both protein and DNA bases are summarized in Table 4. The mean interaction scores for waters bridging protein side chains to DNA bases are 94 and 360 for H_protein-water _and H_DNA-water_, respectively, while the partial Ranks are 1.9 and 1.8.

The previous analyses of bridging waters in protein-ligand systems [44], revealed a global Rank of 4.5 less evenly divided between protein and ligand: the mean partial protein-water Rank was 3.0, while the mean partial ligand-water Rank was 1.5. This difference is probably attributable to the different natures of protein-ligand and protein-DNA interfaces. Proteins, with a more extended and heterogeneous surface characterized by clefts and cavities, usually envelop small ligands, but formation of a protein-DNA complex likely involves winding of the objects together, yielding two more or less comparable surface areas. The HINT score values are also differently distributed in protein-ligand systems compared to protein-DNA systems. In the protein-ligand system [44], H_protein-water _and H_ligand-water _were 307 and 277 HINT score units, respectively, i.e., nearly equal. Here, even in the case of protein side-chain to DNA base, waters interact notably stronger with the DNA (360) than with the protein (94). This is, at first, somewhat surprising, given that the bases are structurally constrained to be planar, while the protein side-chains possess more flexibility and would presumably adopt the most conducive conformation for binding. However, the aromatic groups, present in both pyridine and purine bases, are capable of forming weak hydrogen bonds with water, either by water hydrogen atoms donating to aromatic electron clouds, or by water oxygen atoms accepting from polarized aromatic hydrogens. Thus, nearly all contacts between the nucleic acid bases and the surrounding water molecules are potentially positive. In contrast, hydrophobic protein side-chains would produce numerous unfavorable (negative scored) hydrophobic-polar contacts with water, regardless of the water geometry. Also, structural differences between the two types of interfaces are relevant. The cavities and shallows that bind waters at interfaces in protein-ligand complexes are usually formed by backbone or, more frequently, by charged and polar groups; however, the surface of a protein interacting with a polynucleotide can also be formed by apolar moieties. Thus, even though the number of hydrogen bonds to waters is more equally distributed between the two macromolecules in the protein-DNA case, these waters cannot be enveloped by either the protein or the DNA.

A most interesting consequence of the above results is that water molecules contributing to protein-DNA recognition specificity have a somewhat different set of criteria than those contributing energetically to the complex stability. Visual evaluation indicates that: 1) 54% of waters with nonzero Rank with respect to both macromolecules were involved in interactions with the DNA backbone and thus play a minor role in specificity but are energetically critical for the association. 2) 46% of the waters interact with the nucleotide base; of these, 21% are actually non-bridging, and the remainder (25%) bridge between the base and various features of the protein. Interestingly, only 2–3% of the nucleotide base-bridging waters interact (only) with the protein backbone, so that the vast majority interacts with the protein side-chains and potentially governs binding specificity. It is likely that only these waters forming hydrogen bonds with amino acid side-chains would be involved in recognition of specific nucleic acid sequences, but that accounts for more than 90% of the waters bridging between protein and bases of DNA.

### Character of protein-DNA interactions

**Figure 5:**
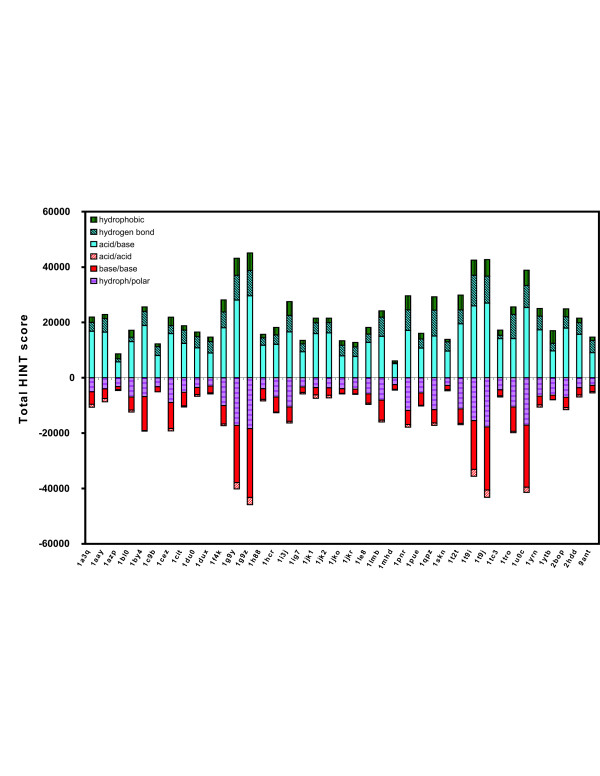
Contributions of the different interaction types participating in the direct protein-DNA association process, as estimated by the HINT force field. The bars are color-coded as indicated in the legend.

Figure 5 illustrates the protein-DNA interactions for the set of 39 complexes in terms of HINT interaction types; i.e., hydrogen-bond, acid/base, hydrophobic, acid/acid, base/base and hydrophobic/polar. As is usually the case in biomolecular associations, the non-covalent forces in protein-DNA association are system-specific and finely balanced [37]. This is evident from Figure 5 where the favorable polar terms (hydrogen-bond and acid/base) are compensated by the base/base, acid/acid and hydrophobic/polar terms. The unfavorable HINT terms represent energy costs such as desolvation that are paid when the complex forms by association of isolated biomolecules. The overall sum of these forces is the binding free energy, generally ranging from -9 to -17 kcal mol^-1 ^[65], and involving electrostatic and van der Waals interactions, hydrogen bonds, ion and water release, complex reorganization due to hydrophobic effects, hydrophobic contacts and other entropy effects [5,36,37,61,65]. While hydrogen bonds (direct and water-mediated) and electrostatic contacts are usually taken into account and considered fundamental in analyses of complex formation and in specific recognition, and are clearly the dominant terms in Figure 5, the other factors related to entropy and hydrophobicity are commonly ignored. We have found that inclusion of hydrophobic terms (favorable hydrophobic-hydrophobic and unfavorable hydrophobic-polar) in scoring models leads to reliable binding free energy predictions in protein-protein [45,46], protein-ligand [29,43] and DNA-ligand [47-49] complexes.

Hydrophobic effects have been proposed to be the major driving forces of protein-DNA association [35,37,66], as this force arises from the burial of non-polar protein surfaces into the DNA binding site. The predominant role of hydrophobicity (i.e., entropy) is supported by calorimetric analyses that reported a negative change in heat capacity upon complex formation [67,68]. On the enthalpic side, the electrostatic term of free energy counteracts binding because favorable charge-charge interactions are often counterbalanced by the highly unfavorable contribution from dehydration of the polar groups [35]. Jayaram's computational analysis of binding [37] also demonstrated that packing and hydrophobic effects favor binding, whereas electrostatic interactions energetically oppose it [41]. However, the negative heat capacity change associated with the formation of specific protein-DNA complexes could not be completely explained by taking into account only hydration effects [14,17,18]. Other contributions, like the conformational changes of both proteins and nucleic acids accounting for 20% of the total ΔC_p _[69-72], the modification of the protonation state of the interacting residues [73] and counterion release [74], have been considered. In particular, even if ion release was generally considered to be favorable for complex formation, several studies demonstrated that the negative contribution from ion-molecule electrostatics, rather than the positive entropy given by the ion reorganization, dominates the salt-dependent solvation effects [36,37]. Furthermore, the ionic interaction with water molecules induces an increased ordering of waters, producing a large negative heat capacity change [14,74].

The HINT analysis in this work allowed examination of the character of interactions contributing to an association without actually parsing them energetically because all atom-atom interactions are evaluated with the same protocol. HINT evaluates not only the electrostatic and van der Waals contributions, but also hydrophobic-related contacts and should be able to evaluate the observation of Mandel-Gutfreund and Margalit [5] that amino acid-nucleotide base recognition is governed by both hydrogen bonds and hydrophobic interactions. Stabilizing hydrophobic contacts, mainly between sugar methylenes and aliphatic or aromatic amino acid side-chains, were estimated to account for 63% of protein-DNA_ribose _contacts [64]. Note that the free energy-based analysis illustrated in Figure 5 is over the entire protein-DNA interaction set (not just protein to ribose). Nevertheless, the hydrophobic/hydrophobic interactions (Figure 5) always contribute favorably to the protein-DNA binding but apparently only to a moderate extent. These contributions are not impacted by unfavorable effects or the presence/absence of bridging waters, and in some cases are the dominant factors in binding after the other terms appear to cancel out. In this sense, hydrophobic contacts and the related hydrophobic effects may represent the main driving force of protein-DNA association, while the electrostatic interactions seem to increase specificity but not affinity. It must be reiterated, however, that this computational analysis tool is probing only the (relatively short range) energetics between pre-formed DNA and protein components of the final, end-state, complex. As such, it does not measure or account for the internal energies of the protein and DNA molecules and the energy involved in conformational changes of these molecules between their unassociated and bound states. The quality of the resulting models, eq. 5, suggests that these and other terms are largely invariant over the data set.

## Conclusion

Water contributes to protein-DNA complex formation in two principal ways. Without water, some of the complexes would be scored as energetically unfavorable. There is an apparent, but interesting, disconnect between water molecules that are significant for DNA-protein recognition having a lower Rank threshold than those critical for accurate free energy calculations. Also, the results above demonstrated that including the energetic contribution from waters at the protein-DNA interface significantly improved the quality of our computational free energy predictions, particularly with only "true" bridging waters. Our criterion, based on the previous analysis of 15 protein-ligand complexes [44], is that only waters characterized by nonzero partial Ranks with each interacting molecule and total Rank of at least 4 are energetically relevant. In effect, a bridging solvent molecule should form a minimum of two strong, well-located hydrogen bonds, with at least one additional favorable contact. Those waters with lower Rank, especially between 3 and 4, are still significant in mapping the energetic landscape for interaction by altering the shape, polarity and surface charge of the DNA or protein, even if they do not directly contribute to the free energy of binding.

This report is the first part in an effort to decode the molecular features leading to protein-DNA recognition. The interaction between these two biomacromolecules is an essential component of the machinery of life. Here we have demonstrated that our modeling experiments, using the empirical HINT free energy forcefield, with a measured incorporation of critical water molecules, gives more than acceptable estimates (± 1.28 kcal mol^-1^) of the free energy of binding. In addition, we have identified a set of traits based on Rank for water molecules that impact binding specificity. The count, orientation and binding strength of this set of water molecules is far more dependent on the chemical nature of the protein amino acid side-chains than on features of the DNA bases. In a forthcoming work, we will explore the specific match-ups of protein amino acid residues and DNA nucleotide bases by their types, with confidence that our computational approach is representative of actual binding free energy, and with these guidelines for the inclusion of relevant water molecules in our models.

## Methods

### Protein-DNA data set

The protein-DNA data set was selected from the available structures in the Protein Data Bank [58]. While there are 123 unique structures in the PDB, many do not have reliable protein-DNA dissociation constants for exactly the same complex, are of poor resolution, and/or have missing residues or bases due to disorder or other experimental factors. The structures of the remaining thirty-nine protein-DNA complexes solved at a resolution better than 2.90 Å (28 complexes at better than 2.50 Å), were retrieved from the PDB and are listed in Table 1. Twenty-one structures are monomeric proteins interacting with double-stranded DNA, while eighteen structures are homodimeric and heterodimeric proteins complexed with palindromic double-stranded DNA. When only the monomeric-single stranded structure was available in the PDB because of crystallographic symmetry, the actual biological complex (i.e., dimeric protein and double-stranded DNA) was obtained from the Nucleic Acid Database . 1jkr and 1jko structures are protein mutants of the 1hcr DNA-native protein complex. Analogously, 1jk1 and 1jk2 are mutants of the 1aay DNA-native complexes, and 1t9j, 1t9i, 1u0c are mutants of 1g9y. Only non-covalent complexes with four or more base pairs in the polynucleotide strand were included in the dataset. PDB files characterized by anomalous DNA structure, non-classical bases or anomalous base-base coupling were not considered. Moreover, only complexes for which published experimental dissociation (K_d_) constants values are available were retained. In particular, to avoid misleading correlations between experimental and computational results, a structure of a particular protein-DNA complex was included in the data set only when the DNA sequence used for the experimental assay was completely coincident with the sequence of the crystallized complex, and when, at least, the same protein domain involved in DNA recognition was used in both binding and crystallographic experiments. When small differences between the DNA sequences used in K_d _determination and crystallization experiments were observed, those complexes were included in our analysis only if the divergent bases were not directly involved in the protein-DNA recognition and association.

### Model building

All complexes were modeled with Sybyl version 7.0 [75]. The structures were carefully checked and corrected for chemically consistent atom and bond type assignment. Hydrogen atoms, not normally detected with common X-ray diffraction techniques, were computationally added, using the Sybyl Biopolymer and Build/Edit menu tools. To avoid steric clashes, added hydrogen atoms were then energy minimized using the Powell algorithm, with a convergence gradient of 0.5 kcal (mol Å)^-1 ^for 1500 cycles, while fixing all heavy atom positions.

### Hydropathic analysis

Hydropathic analyses were carried out with the HINT software [75], using a locally modified version 3.09Sβ [76], as previously reported [29,42-44]. All partition calculations (where atomic HINT constants are assigned based on LogP_o/w_) were performed using the dictionary option for both proteins and nucleic acid sequences [77]. In this work ionization states of neither protein residues nor DNA nucleotides were modified, i.e., keeping the default protonation models (ca. pH 7) of Sybyl. Because the interactions between proteins and nucleic acids are mainly electrostatic and H-bond based, the 'essential' option, which treats only the polar hydrogen atoms explicitly, was chosen as partition mode. A new HINT option that corrects the S_i _terms for backbone amide nitrogens and hydrogens [78] by adding 20 Å^2 ^was used in this study. This correction improves the relative energetics of inter- and intra-molecular hydrogen bonds involving backbone amides.

### Energetic contribution of water molecules

Water molecules crystallographically placed at the protein-DNA interface in a 4 Å range were automatically optimized and scored, using the "optimize bridging waters" and the "water accounting" options, implemented in the 3.09Sβ HINT version. For all of the succeeding calculations, each water was treated as an individual static molecule, and no statistical mechanical averaging on dynamics simulation trajectories were performed. During HINT optimization, the crystallographically-determined oxygen atom is allowed to translate at most 0.1 Å around its original position. HINT scores involving water are calculated as if each water molecule is a "ligand" interacting with the surrounding biomolecules acting in concert as a "receptor". Next, the "optimize water network" option was applied on crystallographic waters within 4 Å of both atoms of the protein and atoms of DNA using the geometry-based Rank algorithm [44,60]. Rank is able to predict the weighted number of potential hydrogen bonds formed by each water molecule with both the protein and the DNA sequence. During the optimization process the water hydrogen atoms are allowed to adopt all possible positions in order to maximize hydrogen bonds and acid/base interactions, and to minimize unfavorable hydrophobic/polar or acid/acid contacts; i.e., the process is exhaustive. Only waters exhibiting Rank values greater than 0 with both protein and nucleic acid are considered bridging water molecules [44]. Waters forming hydrogen bonds with only the protein, the DNA or neither are considered as waters of solvation that are not involved in the binding event and presumed to be not essential to the energetics of complex formation. Therefore, for each analyzed complex, the contribution given by waters characterized by Rank > 0 was calculated and added to the protein-DNA HINT score, i.e., H_TOT _= H_protein-DNA _+ H_protein-water _+ H_DNA-water_. Even though the Rank algorithm allows each water molecule to act as donor with at most two hydrogen bond acceptors and as acceptor with at most two hydrogen bond donors, Rank should be interpreted only loosely as a count of hydrogen bonds. In previous analyses performed on protein-ligand complexes [44], Rank greater than four was associated with very locked and stable water molecules. Thus, in this work, bridging waters with total Rank ≥ 4 were identified for special consideration (see Results and Discussion).

### Identification of water molecules mediating specific protein-DNA recognition

Some water molecules are specific mediators of recognition between protein and DNA. To isolate specific interactions between protein and base atoms, the phosphate and ribose groups were excluded from the HINT partition. Again, water molecules found in a 4 Å range at the protein-DNA interface with Rank > 0 were optimized, scored and Ranked only with respect to protein residues and DNA bases. These waters, potentially significant for specific recognition and association, were classified as bridging or not bridging. Another constraint is that bridging waters must mediate interactions between groups that are too far to contact each other otherwise. The bridging waters were divided into three different classes: (I) waters bridging DNA bases and protein amino acid residue side-chains, (II) waters bridging DNA bases and the protein backbone, and (III) waters bridging DNA bases and both protein side-chain and backbone atoms. Specific mean HINT score and Ranks were determined for each category, paying particular attention to side chain bridging waters, the only that should be able to mediate specific recognition. HINT score and Rank diagnostic of the three classes were calculated in order to identify essential water molecules in new protein-DNA complexes.

## Authors' contributions

FS, CB and AMa built and optimized the protein-DNA-water molecular models. FS designed and performed the hydropathic calculations and performed data/statistical analysis/reduction. PC and AMo designed and coordinated the study. GEK created custom modeling software and suggested data analysis protocols. The manuscript was written by FS, PC, GEK and AMo. All authors read and approved the final manuscript.

## Figures and Tables

**Table 1:** Classification data for the 39 protein-DNA complexes used in this study.

**PDB code**	**Name**	**classification**	**Family**	**DNA-binding motif**	**C.A.T.H**.	**Structure ref**.
1a3q	NF kB p52	Transcr. regulator	Rel homology region	other	Mixed ^b^	[79]
1aay	Zif268 zinc finger	Transcr. regulator	ββα-zinc finger	Zinc coordinating group	3.30.160.60	[80]
1azp	Sac7D	DNA binding	Hyperthermophyle DNA-BP	β-sheet group	2.40.50.40	[81]
1bl0	MarA	Transcr. regulator	AraC transcriptional activator	Helix-turn-helix	1.10.10.60	[82]
1by4	Retinoic acid receptor rxr-alpha	Transcr. regulator	Nuclear receptor	Zinc coordinating group	3.30.50.10	[83]
1c9b	TF IIB	Transcr. initiator	TF IIB	Helix-turn-helix	1.10.472.10	[84]
1cez	T7 RNA polymerase	Enzyme	T7 RNA polymerase	DNA/RNA polymerases	Mixed ^b^	[85]
1cit	Orphan nuclear receptor NGFI-B	Transcr. regulator	Nuclear receptor	Zinc coordinating group	3.30.50.10	[86]
1du0	Engrailed homeodomain (q50a)	Transcr. regulator	Homeodomain	Helix-turn-helix	1.10.10.60	[87]
1dux	Elk-1	Transcr. regulator	Ets domain	Winged HTH	1.10.10.10	[88]
1f4k	Replication terminator protein	DNA binding	Replication terminator protein	Winged HTH	1.10.10.10	[89]
1g9y ^a^	Homing endonuclease I-CreI	Enzyme	Homing endonuclease	Homing endonuclease-like	3.10.28.10	[90]
1g9z ^a^	Homing endonuclease I-CreI	Enzyme	Homing endonuclease	Homing endonuclease-like	3.10.28.10	[90]
1h88	Myb proto-oncogene protein	Transcr. regulator	Homeodomain	Helix-turn-helix	1.10.10.60	[91]
1hcr	Hin-recombinase	Enzyme	Hin-recombinase	Helix-turn-helix	1.10.10.60	[92]
1i3j	Homing endonuclease I-TevI	Enzyme	DNA-binding domain of intro endonuclease	DNA-binding domain of intro endonuclease	3.30.60.40	[93]
1ig7	Homeotic protein Msx-1	Transcr. regulator	Homeodomain	Helix-turn-helix	1.10.10.60	[94]
1jk1	Zif268 d20a mutant	Transcr. factor	ββα-zinc finger	Zinc coordinating group	3.30.160.60	[95]
1jk2	Zif268 d20a mutant	Transcr. factor	ββα-zinc finger	Zinc coordinating group	3.30.160.60	[95]
1jko	Hin recombinase	Enzyme	Homeodomain	Helix-turn-helix	1.10.10.60	[96]
1jkr	Hin recombinase	Enzyme	Homeodomain	Helix-turn-helix	1.10.10.60	[96]
1le8	3A Mating-type protein a-1	Transcr. regulator	Homeodomain	Helix-turn-helix	1.10.10.60	[97]
1lmb	Lambda repressor	Transcr. regulator	Repressor	Helix-turn-helix	1.10.260.40	[98]
1mhd	Smad3	Transcr. regulator	Smad Mh1 Domain	Smad Mh1 Domain	3.90.520.10	[99]
1pnr	Purine repressor	Transcr. regulator	LacI repressor	Helix-turn-helix	1.10.260.40	[100]
1pue	TF PU.1	Transcr. factor	Ets domain	Winged HTH	1.10.10.10	[101]
1qpz	Purine repressor	Transcr. regulator	LacI repressor	Helix-turn-helix	1.10.260.40	[102]
1skn	Transcription factor skn-1	Transcr. factor	Transcription factor skn-1	Other α-helix group	1.10.880.10	[103]
1t2t	Homing endonuclease I-TevI	Enzyme	DNA-binding domain of intro endonuclease	DNA-binding domain of intro endonuclease	3.30.60.40	[104]
1t9i ^a^	Homing endonuclease I-CreI (d20n)	Enzyme	DNA-binding domain of intro endonuclease	DNA-binding domain of intro endonuclease	3.10.28.10	[105]
1t9j ^a^	Homing endonuclease I-CreI (q47e)	Enzyme	DNA-binding domain of intro endonuclease	DNA-binding domain of intro endonuclease	3.10.28.10	[105]
1tc3	Transposase Tc3a1-65	Enzyme	Transposase	Helix-turn-helix	1.10.10.60	[106]
1tro	Tryptophane repressor	Transcr. regulator	Tryptophane repressor	Helix-turn-helix	1.10.1270.10	[107]
1u0c ^a^	Homing endonuclease I-CreI (y33c)	Enzyme	DNA-binding domain of intro endonuclease	DNA-binding domain of intro endonuclease	3.10.28.10	[108]
1yrn	Mating-type protein a-1	Transcr. regulator	Homeodomain	Helix-turn-helix	1.10.10.60	[109]
1ytb	TATA box binding protein	Transcr. initiator	TATA box binding protein	β-sheet group	3.30.310.10	[110]
2bop	Bovine papillomavirus-I E2	Transcr. regulator	bovine papillomavirus-I E2	Other α-helix group	3.30.70.330	[111]
2hdd	Engrailed homeodomain (q50k)	Transcr. regulator	Homeodomain	Helix-turn-helix	1.10.10.60	[112]
9ant	Antennapedia protein	Transcr. regulator	Homeodomain	Helix-turn-helix	1.10.10.60	[113]

^a ^Homing endonuclease I-CreI-DNA complexes (see text for more information).

^b ^The domain arrangements of the protein portions interacting with the DNA are identified by multiple C.A.T.H. codes.

**Table 2:** Structural, experimental dissociation and calculated HINT score data for the 39 protein-DNA complexes.

					**HINT scores for protein-DNA complexes**			
					
**PDB code (Resolution, Å)**	**Total water count**	**pK_d _(M)**	**K_d _ref**	**ΔG° (kcal mol^-1^)**	**H_protein-DNA_**	**H_P-D-W _**^*a *^(water count)	**H_P-D-W _**(R > 0)^*b *^(water count)	**H_P-D-W _**(R≥4)^*c *^(water count)
1a3q (2.10)	785	10.82	[79]	-14.71	11257	22843 (39)	19954 (28)	12461 (2)
1aay (1.60)	148	9.90	[114]	-13.46	14166	36884 (47)	36300 (43)	20454 (11)
1azp (1.60)	132	6.81	[115]	-9.26	4045	9182 (16)	8400 (12)	5225 (4)
1bl0 (2.30)	144	7.70	[116]	-10.46	4742	6513 (15)	6294 (12)	5143 (4)
1by4 (2.10)^*d*^	120	6.46	[83]	-8.78	6328	17213 (24)	14606 (19)	10407 (7)
1c9b (2.65)^*d*^	101	7.35	[117]	-9.99	7122	14402 (20)	13101 (14)	7355 (1)
1cez (2.40)	471	6.33	[118]	-8.60	2697	8969 (17)	8125 (12)	4807 (4)
1cit (2.70)	38	9.00	[119]	-12.23	8239	12266 (9)	12347 (8)	9973 (4)
1du0 (2.00)	103	9.72	[120]	-13.21	9793	15802 (37)	16295 (24)	12380 (5)
1dux (2.10)	129	10.07	[121]	-13.69	8837	18746 (24)	18768 (20)	13227 (7)
1f4k (2.50)	96	10.70	[89]	-14.54	10836	18458 (20)	17928 (18)	14753 (8)
1g9y (2.05)	435	10.00	[105]	-13.59	2991	37385 (102)	33546 (83)	16470 (30)
1g9z (1.80)	857	8.70	[122]	-11.83	-747	32408 (117)	31561 (93)	10064 (25)
1h88 (2.80)	25	7.45	[91]	-10.13	7335	8677 (3)	8436 (2)	7335 (0)
1hcr (1.80)	16	7.47	[96]	-10.15	5553	6173 (4)	6173 (4)	5553 (0)
1i3j (2.20)	185	8.02	[123]	-10.90	11217	21035 (37)	21381 (30)	13263 (6)
1ig7 (2.20)	153	8.70	[124]	-11.83	7656	19284 (34)	10839 (27)	10839 (6)
1jk1 (1.90)	136	10.59	[95]	-14.39	14098	38199 (53)	35286 (40)	18768 (7)
1jk2 (1.65)	145	10.37	[95]	-14.09	14283	35196 (51)	33716 (42)	19239 (7)
1jko (2.24)	13	7.03	[96]	-9.55	7571	7731 (1)	7731 (1)	7732 (1)
1jkr (2.28)	2	6.08	[96]	-8.27	6787	6787 (0)	6787 (0)	6787 (0)
1le8 (2.30)	102	6.66	[97]	-9.05	8571	12904 (26)	12679 (18)	8990 (2)
1lmb (1.80)	140	9.00	[98]	-12.23	8191	19046 (37)	16445 (31)	10154 (6)
1mhd (2.80)	24	6.93	[99]	-9.42	1607	1660 (2)	1660 (2)	1629 (1)
1pnr (2.70)	92	8.47	[125]	-11.51	11760	14090 (6)	14090 (6)	11760 (0)
1pue (2.10)^*d*^	61	6.85	[126]	-9.30	5823	11403(18)	10461 (14)	6659 (1)
1qpz (2.50)	184	8.59	[102]	-11.67	12015	16625 (12)	16877 (12)	13527 (4)
1skn (2.50)	28	9.00	[103]	-12.23	9158	10976 (6)	10976 (6)	9618 (2)
1t2t (2.50)	72	8.28	[104]	-11.25	12893	16500 (13)	15693 (7)	14360 (4)
1t9i (1.60)	656	8.74	[105]	-11.89	6895	49015 (122)	43140 (98)	18371 (31)
1t9j (2.00)	189	9.22	[105]	-12.54	-449	27873 (81)	24965 (70)	8215 (22)
1tc3 (2.45)	49	7.10	[127]	-9.65	10246	13475 (9)	13390 (6)	10720 (1)
1tro (1.90)	572	9.30	[128]	-12.64	5739	22606 (60)	22434 (46)	10568 (12)
1u0c (2.50)	90	8.23	[108]	-11.19	-2591	12569 (37)	7561 (30)	3622 (14)
1yrn (2.50)	58	10.00	[129]	-13.59	14429	22584 (25)	22846 (24)	17871 (9)
1ytb (1.80)	513	8.40	[130]	-10.06	9035	16607 (32)	17529 (23)	9996 (1)
2bop (1.70)	121	9.40	[131]	-12.77	13400	22452 (33)	22254 (24)	14692 (2)
2hdd (1.90)	183	11.06	[120]	-15.03	14613	31840 (46)	30951 (39)	18688 (9)
9ant (2.40)	26	8.80	[132]	-11.96	9192	12971 (9)	13049 (8)	11711 (5)

^*a *^H_P-D-W _= H_protein-DNA _+ H_protein-water _+ H_DNA-water_. Only the contributions of water molecules in a 4 Å range at the protein-DNA interface are considered.

^*b *^Only waters with nonzero Ranks with respect to both protein and DNA are included in H_P-D-W_.

^*c *^Only waters with nonzero Ranks with respect to both protein and DNA and total Rank ≥ 4 are included in H_P-D-W_.

^*d *^The PDB files report the structures as two equivalent protein-DNA complexes. The second complexes were removed and only waters surrounding the first in a 8 Å sphere were considered.

**Table 3:** Number, mean HINT scores and mean Ranks of waters found at the protein-DNA interface.

	**Water count**	**Mean HINT score**			**Mean Rank**		
		
		**H_protein-water_**	**H_DNA-water_**	**H_TOTAL_**	**R_protein-water_**	**R_DNA-water_**	**R_TOTAL_**
**All within 4 Å**	1244	-22	355	333	1.6	1.4	3.0
**global Rank > 0, partial Ranks > 0**	996	-10	388	378	1.8	1.6	3.4
**global Rank ≥ 4, partial Ranks > 0**	261	21	432	452	3.0	2.0	5.0

**Table 4:** Number, mean HINT scores and mean Ranks of waters initially classified by role.

		**Water count**	**Mean HINT score**			**Mean Rank**		
			**H_protein-water_**	**H_DNA-water_**	**H_TOTAL_**	**R_protein-water_**	**R_DNA-water_**	**R_TOTAL_**
**All H_2_O interacting with DNA backbone**		535	-36	426	390	1.9	1.3	3.2
**All H_2_O interacting with nucleotide base**		461	32	324	356	1.7	1.6	3.3
**All non-bridging H_2_O**		212	-17	281	264	1.6	1.2	2.8
**All H_2_O bridging nucleotide base and protein**		249	71	361	432	1.9	1.8	3.7
**H_2_O bridging nucleotide base and protein's**	**side-chain**	218	94	360	453	1.9	1.8	3.7
	**backbone**	20	-120	400	280	1.6	1.9	3.5
	**backbone & side-chain**	11	36	310	346	2.8	1.7	4.6
